# Interconnection of Meaning and Purpose in Life, Sense of Belonging and Social Engagement in People Living with HIV

**DOI:** 10.1093/geroni/igaf122.503

**Published:** 2025-12-31

**Authors:** Atami De Main, Marshall J Glesby, Carrie Johnston, Mark Brennan Ing, Eugenia L Siegler

**Affiliations:** Weill Cornell Medicine, New York, New York, United States; Weill Cornell Medicine, New York, New York, United States; Weill Cornell Medicine, New York, New York, United States; Hunter College, New York, New York, United States; Weill Cornell Medicine, New York, New York, United States

## Abstract

Despite advancements in medical treatment for HIV/AIDS, people living with HIV (PWH) still encounter health, social and structural vulnerabilities. Understanding factors that contribute to PWH’s mental health and well-being become increasingly important. Psychosocial factors — meaning and purpose in life, sense of belonging and social engagement — play a protective role against negative health outcomes. Our study aimed to examine the associations among these factors, using items from the Brief Multidimensional Measure of Religiousness and Spirituality scale, in older PWH (n = 349, age range=50-84) from the Research on Older Adults with HIV (ROAH 2.0) study at the Weill Cornell Campus in New York City. Hierarchical regression analyses revealed that lower levels of loneliness, fewer depressive symptoms, lower number of chronic conditions and higher engagement in social activities and media-based activities were significantly associated with strong general sense of belonging in older PWH (R2adj=0.34, F(10, 269) = 15.61, p<.001), strong feelings of being part of a community (R2adj=0.35, F(10, 262) = 15.56, p<.001) and high meaning and purpose in life (R2adj=0.34, F(9, 281) = 17.49, p<.001). Additionally, PWH experiencing financial strain and those living alone reported lower general sense of belonging (F(6, 286) = 2.99, p<.001, feelings of being part of a community F(6, 280) = 1.91, p<.001) and meaning and purpose in life F(5, 297) = 2.50, p = 0.03). These findings underscore the importance of psychosocial indicators in promoting the mental health and well-being of older PWH and highlight the need for interventions to enhance social and community engagement in this population.

